# Dynamic changes in clinical biomarkers of cardiometabolic diseases by changes in exercise behavior, and network comparisons: a community-based prospective cohort study in Korea

**DOI:** 10.4178/epih.e2023026

**Published:** 2023-02-16

**Authors:** JooYong Park, Jaesung Choi, Ji-Eun Kim, Sang-Min Park, Joo-Youn Cho, Daehee Kang, Miyoung Lee, Ji-Yeob Choi

**Affiliations:** 1 Department of Big Data Medical Convergence, Eulji University, Seongnam, Korea; 2 Department of Biomedical Sciences, Seoul National University Graduate School, Seoul, Korea; 3 Institute of Health Policy and Management, Seoul National University Medical Research Center, Seoul, Korea; 4 Department of Family Medicine, Seoul National University Hospital, Seoul National University College of Medicine, Seoul, Korea; 5 Department of Clinical Pharmacology and Therapeutics, Seoul National University College of Medicine and Hospital, Seoul, Korea; 6 Department of Preventive Medicine, Seoul National University College of Medicine, Seoul, Korea; 7 College of Physical Education and Sport Science, Kookmin University, Seoul, Korea

**Keywords:** Exercise, Cardiometabolic diseases, Clinical biomarkers, Network

## Abstract

**OBJECTIVES:**

Lifestyles, including exercise behaviors, change continually over time. This study examined whether the clinical biomarkers (CBs) related to cardiometabolic diseases (CMDs) and their relationships differed with changes in exercise behavior.

**METHODS:**

The Ansan-Ansung cohort study (third to fifth phases; n=2,668) was used in the current study. Regular exercise behavior was investigated using a yes/no questionnaire. Changes in exercise behavior were classified into 4 groups: Y-N, N-Y, Y-Y, and N-N, with “Y” indicating that a participant regularly engaged in exercise at a given time point and “N” indicating that he or she did not. Fourteen CBs related to CMDs were used, and the associations between changes in exercise behavior and relative changes in CBs were examined. CB networks were constructed and topological comparisons were conducted.

**RESULTS:**

Y-N was associated with increases in fasting blood sugar and insulin levels in men, and increased total cholesterol and low-density lipoprotein cholesterol levels in women. Meanwhile, N-Y was inversely associated with body fat percentage, visceral fat percentage, fasting insulin, and triglyceride level. Waist circumference played a central role in most networks. In men, more edges were found in the N-Y and Y-Y groups than in the N-N and Y-N groups, whereas women in the N-Y and Y-Y groups had more edges than those in the N-N and Y-N groups.

**CONCLUSIONS:**

Consistent exercise or starting to engage in regular exercise had favorable effects on CBs related to CMDs, although their network patterns differed between the sexes.

## GRAPHICAL ABSTRACT

[Fig f4-epih-45-e2023026]

## INTRODUCTION

Cardiometabolic diseases (CMDs), defined as a cluster of abdominal obesity, hypertension, dyslipidemia, hyperinsulinemia, and glucose intolerance, can lead to cardiovascular disease and type 2 diabetes [1]. Therefore, risk factors for CMDs include various clinical biomarkers (CBs) such as blood pressure, obesity-related indices, glucose levels, and lipid profiles. These CBs may contribute to the incidence of CMDs by interacting with each other in complex ways.

Physical activity (PA) and exercise, which are modifiable lifestyle factors, have well-established favorable effects on health in several epidemiological studies [2-4]. However, most studies used PA or exercise variables at a single time point and examined their associations with health outcomes. Lifestyles, including PA and exercise behaviors, change continually over time. Therefore, it is necessary to study the effects of changes in PA or exercise behaviors on health.

A few studies have examined the association between changes in PA and health. Men who improved their physical fitness levels from unfit to fit showed lower all-cause mortality than those who remained physically unfit [5]. Men who increased their level of leisure-time physical activity (LTPA) showed reduced all-cause mortality in their 80s compared to those who maintained a low-medium level of PA or who decreased their PA level from high to low-medium [6]. Likewise, sedentary women who became active showed lower mortality than those who remained sedentary [7]. Increased PA levels or becoming active decreased the risk of metabolic syndrome [8,9]. Moreover, decreased PA was associated with higher cholesterol levels [10], while increasing PA reduced cholesterol, glucose levels, blood pressure, and waist circumference [11-13].

However, these studies reported only one-to-one relations for each outcome variable or risk factor and did not discuss or suggest biological processes, such as interactions among variables or potential mechanisms. Here, we examined whether the effects on CMD-related CBs differed according to patterns or changes in regular exercise behavior. To further understand the potential biological mechanisms, we also used topological network comparisons to analyze the relationships among CBs by patterns or changes in regular exercise.

## MATERIALS AND METHODS

### Study population

**Figure 1.**
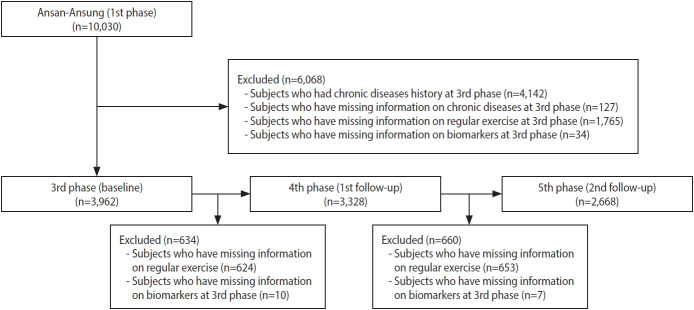
Inclusion and exclusion criteria of the study population in community-based cohort study (Ansan-Ansung cohort study).

This study used the community-based Ansan-Ansung cohort study, which is part of the Korean Genome and Epidemiology Study. Subjects who lived in Ansan or Ansung (Gyeonggi Province) and who were aged 40–69 were recruited in 2001–2002 (n= 10,030). Follow-up examinations took place biennially. Details of the study design, data collection methods, and other information have been described elsewhere [14]. The third phase of the cohort study, conducted in 2005–2006, was considered the baseline for the current study because the regular exercise questionnaire was consistent thereafter. We used data from the third phase (baseline) to the fifth phase (second follow-up) in the current study. After excluding those who had a history of any cancer, cardiovascular disease, type 2 diabetes, hypertension, or dyslipidemia; for whom disease history information was missing; for whom regular exercise information was missing; and for whom clinical parameter values were missing, 3,962 subjects were included in the baseline analyses. Subsequently, subjects were excluded if data regarding clinical parameters or regular exercise from the fourth to fifth phases of the study were missing; thus, 2,668 were included in the final analyses (Figure 1).

### Patterns and changes in exercise behavior

Participation in regular exercise was investigated using a unified question from the third phase (baseline in this study) of the AnsanAnsung cohort study. The subjects answered “yes” or “no” to the question, “Do you regularly exercise enough to make your body sweat?” By combining answers from the surveys for the third to fifth phases, we classified the subjects into 2 consistent behavior groups, “no exercise consistently (N-N)” and “consistent participation in regular exercise (Y-Y),” as well as 2 changed behavior groups, “change to exercise behavior (N-Y)” and “change to no exercise behavior (Y-N)” (Supplementary Material 1).

### Changes in clinical biomarkers

CBs related to CMDs obtained through physical examination and clinical blood tests were used in this study. Blood pressure was measured in both arms with patients in a seated position, and the average value was recorded as systolic blood pressure (mmHg) and diastolic blood pressure (mmHg). Waist circumference was measured horizontally at the midpoint between the iliac crest and the lowest rib, and hip circumference was measured horizontally at the widest part of the hip. Waist and hip circumferences were measured in centimeters to 1 decimal place. The waist-to-hip ratio (WHR) was obtained from the measured waist and hip circumferences. Obesity-related biomarkers such as body fat percentage (%), visceral fat percentage (%), and obesity degree (%) were obtained by an InBody device (Biospace, Seoul, Korea). Diabetes-related biomarkers, including fasting blood sugar (mg/dL), hemoglobin A1c (HbA1c, %), and fasting insulin (μIU/mL), as well as lipid-related biomarkers, including total cholesterol (mg/dL), high-density lipoprotein (HDL) cholesterol (mg/dL), low-density lipoprotein (LDL) cholesterol (mg/dL), and triglyceride (mg/dL) levels, were measured from blood samples collected after at least an 8-hour fast. Fourteen CBs were included in this study. The relative change in CBs was calculated by dividing the difference between each CB at follow-up and baseline by the baseline CB and then multiplying by 100: [(CB at follow-up–CB at baseline)/CB at baseline]× 100.

### Covariates

To examine the characteristics of the study population, we used socio-demographic information, such as education level, income, marital status, current occupation, smoking status, and drinking habit, which was gathered via questionnaire during the third phase (baseline). Educational level was categorized as ≤ middle school, high school, and ≥ college. Monthly income was categorized as ≤ 2,000,000 Korean won (KRW), 2,000,000-4,000,000 KRW, and ≥ 4,000,000 KRW. Current occupation was classified into 4 groups: office workers, manual workers, unemployed/homemakers, and military/other. Participants were classified by smoking status as non-smokers (never), former smokers, and current smokers and by drinking behavior as non-drinkers (never), former drinkers, and current drinkers. Body mass index (BMI) was calculated using the measured height and weight (kg/m^2^).

### Statistical analysis

The Wilcoxon rank-sum test and chi-square test were used to analyze differences in basic characteristics and CB distribution between men and women. The association between participation in regular exercise at baseline and CBs at baseline was estimated using a generalized linear regression model adjusted for age, education level, income, marital status, occupation, BMI, smoking status, and drinking behavior. The least-squares means method, adjusted for age, was used to estimate average changes in CBs for each of the 4 groups defined according to the patterns and changes in regular exercise behavior (“no exercise consistently [N-N]” and “consistent participation in regular exercise [Y-Y],” and the 2 changed behavior groups, “change to exercise behavior [N-Y]” and “change to no exercise behavior [Y-N]”). A generalized linear regression model was used to examine the associations between these patterns and changes in CB adjusted for age. The false discovery rate p-value was calculated to correct for multiple tests.

To construct a CB network assuming that the effect of patterns and changes in regular exercise from baseline to follow-up would influence these CBs at follow-up, partial correlation matrices of CBs at follow-up were calculated by adjusting for age in each of the 4 groups defined by their regular exercise behavior [15]. When the partial correlation coefficient |r| was > 0.3 and the p-value was < 0.05, the relationships among CBs were visualized as a network using Cytoscape version 3.7.2 [16]. CBs were presented generally as nodes, and some CBs were presented as rhombuses when their changes in value were associated with significant changes in regular exercise behavior. The node color indicates changes in CB (red: least-square means of CB changes are significant and the values are positive). The edges indicate a significant partial correlation between nodes. The red and blue edges indicate positive and negative correlations, respectively.

All statistical analyses were conducted using SAS version 9.4 (SAS Institute Inc., Cary, NC, USA) and R version 4.0.0 (R Foundation for Statistical Computing, Vienna, Austria), and network analyses and topological comparisons were implemented using Cytoscape version 3.7.2 (Cytoscape Consortium, San Diego, CA, USA).

### Ethics statement

All participants provided informed consent, and the study was approved by the Institutional Review Board of Seoul National University Hospital, Seoul, Korea (IRB No. E-2009-008-1153).

## RESULTS

Except for regular exercise, the subjects’ characteristics differed significantly between men and women (Supplementary Material 2). Significant differences between men and women were also observed in most CBs at baseline (Supplementary Material 3); therefore, all analyses were performed separately for men and women.

The characteristics of study participants according to regular exercise at baseline did not differ between men and women. Both men and women were more likely to exercise regularly when they were of higher education level, higher income, overweight, office workers, or unemployed/homemakers. Additionally, men who were non-smokers and women who drank alcohol were more likely to exercise regularly (Table 1).

Associations between CB and participation in regular exercise at baseline are shown in Table 2. Waist circumference, WHR, and visceral fat percentage were negatively associated with regular exercise, whereas HDL was negatively associated with regular exercise in both men and women. Additionally, total cholesterol and LDL levels were positively associated with regular exercise, while fasting insulin was negatively associated with regular exercise in women only.

According to their respective patterns of participation in regular exercise, the characteristics of study subjects from the third phase (baseline) to the fifth phase (second follow-up) were similar to those at baseline (Supplementary Material 4). Men who engaged in regular exercise consistently from baseline to follow-up showed a smaller increase in waist circumference and fasting blood sugar than those who did not exercise consistently, while negative associations were observed between them (waist circumference: β = -0.9966, p = 0.006; fasting blood sugar: β = -0.9966, p= 0.006) (Table 3). When men who exercised regularly at baseline stopped exercising at follow-up, a further increase in fasting blood sugar was observed compared to the values in those who engaged in regular exercise consistently (Table 3). Meanwhile, favorable changes in body fat percentage, visceral fat percentage, fasting insulin level, and triglyceride level were observed when men who did not exercise regularly at baseline started exercising, compared to those who did not engage in regular exercise consistently (Table 3). Total cholesterol and LDL levels were lower in women who engaged in regular exercise consistently than in those who did not (Table 3). However, those CBs increased more when women who exercised regularly at baseline stopped exercising than in those who continued exercising regularly (Table 3). Smaller increases in triglyceride levels were noted in women and men who changed their exercise behavior, even when they did not regularly exercise at baseline, than in those who never exercised consistently (Table 3).

**Figure 2.**
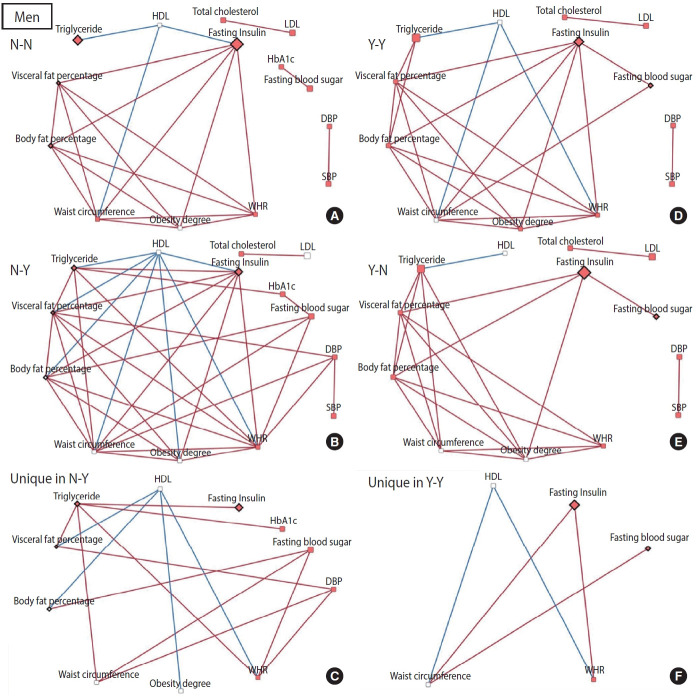
Networks of clinical biomarkers according to exercise behavior in men. Networks were constructed based on partial correlation coefficients adjusted for age (|*r*|>0.3 and p-value <0.05). (A) Network of clinical biomarkers in men who did not regularly exercise consistently (14 nodes, and 21 edges). (B) Network of clinical biomarkers in men who changed to exercise behavior (14 nodes, and 36 edges). (C) Unique network structure in men who changed to exercise behavior. (D) Network of clinical biomarkers in men who did regular exercise consistently (13 nodes, and 24 edges). (E) Network of clinical biomarkers in men who changed to non-exercise behavior (13 nodes, and 21 edges). (F) Unique network structure in men who did regular exercise consistently. Rhombus nodes (◇): the changes in clinical biomarkers were significantly associated with changes in regular exercise. Red node color: LSmeans of clinical biomarker changes are significant and the values are positive. Red edges: positive correlations. Blue edges: negative correlations. LSMean, least-squares mean; SBP, systolic blood pressure; DBP, diastolic blood pressure; HbA1c, hemoglobin A1c; HDL, high-density lipoprotein; LDL, low-density lipoprotein; WHR, waistto-hip ratio.

**Figure 3.**
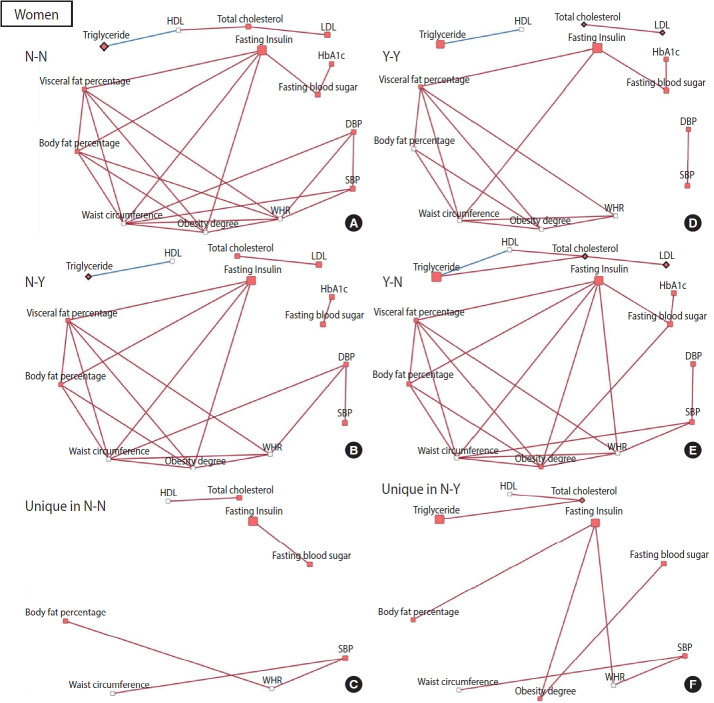
Networks of clinical biomarkers according to exercise behavior in women. Networks were constructed based on partial correlation coefficients adjusted for age (|*r*|>0.3 and p-value <0.05). (A) Network of clinical biomarkers in women who did not regularly exercise consistently (14 nodes, and 24 edges). (B) Network of clinical biomarkers in women who changed to exercise behavior (14 nodes, and 19 edges). (C) Unique network structure in women who did not regular exercise consistently. (D) Network of clinical biomarkers in women who did regular exercise consistently (14 nodes, and 16 edges). (E) Network of clinical biomarkers in women who changed to non-exercise behavior (13 nodes, and 24 edges). (F) Unique network structure in women who changed to non-exercise behavior. Rhombus nodes (◇): the changes in clinical biomarkers were significantly associated with changes in regular exercise. Red node color: LSmeans of clinical biomarker changes are significant and the values are positive. Red edges: positive correlations. Blue edges: negative correlations. LSMean, leastsquares mean; SBP, systolic blood pressure; DBP, diastolic blood pressure; HbA1c, hemoglobin A1c; HDL, high-density lipoprotein; LDL, low-density lipoprotein; WHR, waist-to-hip ratio.

The relationships among CBs also differed depending on the pattern and changes in exercise behavior. In men, more edges appeared in the network of the group that started exercising than in the network of the group that consistently did not exercise, and fewer edges appeared in the network of the group that stopped exercising than in the network of the group that participated in regular exercise consistently (Figure 2). Conversely, for women, fewer edges appeared in the network of the group that started exercising than in the network of the group that did not exercise consistently, and there were more edges in the network of the group that quit exercising than in the network of the group that continued exercising regularly (Figure 3). In most networks, obesity-related CBs such as waist circumference, WHR, visceral fat percentage, body fat percentage, and obesity degree had more edges linked than the others, meaning that these clinical variables are more likely to play a central role in the network. In topological comparisons of networks according to changes in exercise behavior, fasting blood sugar or fasting insulin, which were significantly associated with changes in exercise behavior in men, remained in the networks that formed unique structures in men who exercised consistently and in men who started exercising. Meanwhile, significant results in women’s lipid-related biomarkers, including total cholesterol, HDL, and triglyceride levels, remained when the networks of women were compared topologically according to changes in exercise behavior (Figures 2 and 3).

## DISCUSSION

This study examined the effects over time of changes in exercise behavior upon changes in CBs. We showed how the relationships among CBs change according to changes in exercise patterns. When people quit exercising, the fasting blood glucose level in men and the lipid-related biomarkers in women increased. Conversely, when people who did not exercise started exercising regularly, body fat-related biomarkers, fasting insulin, and triglycerides changed favorably. It is noteworthy that triglyceride levels increased less when both men and women who had not exercised previously started exercising. Via network analysis, a topological difference in the relationships among CBs was observed depending on the pattern and change in exercise behavior.

The associations between changes in exercise behavior and changes in CBs observed in the present study support those of previous studies. When both men and women increased their LTPA levels, the increase in total cholesterol was smaller than in those with decreased LTPA levels [10]. Decreased blood glucose levels were observed in men with increased PA intensity and levels in a previous study [6]. Balkau et al. [12] also reported that fasting insulin and glucose levels decreased in men as their athletic activity increased. Compared to people consistently involved in moderate to high levels of PA, those who consistently participated in low levels of PA experienced an increase in waist circumference. An even greater increase in waist circumference was observed in those whose PA levels decreased [13]. Moreover, increased PA was inversely associated with total cholesterol and LDL cholesterol, and marginally with triglyceride levels [13].

In addition to these consistent results, through a topological comparison, we proposed a potential mechanism by showing how the relationships among CBs differed according to the pattern and changes in exercise behavior. More edges were noted when men continued to engage in regular exercise or men who were sedentary became physically active. An increase in edges appeared around fasting insulin and blood sugar levels. When men continued to exercise or started exercising, the relationships among CBs appeared to be stronger; in particular, the mechanistic role of blood sugar-related markers increased. In contrast, more edges were observed in the networks of women who did not exercise consistently or who quit exercising. These findings suggest that when women do not exercise, relationships among clinical indicators are strengthened and the mechanistic role of lipid-related biomarkers is particularly prominent.

The differences in results between men and women might be due to differences in exercise participation characteristics, motivation factors, and biological metabolism. Men generally perform longer exercise sessions at higher intensity than women; moreover, the exercise types and frequencies differ between the sexes [17-19]. Our data demonstrated that the total exercise time was higher in men, while the exercise frequency was higher in women. As for exercise type, men mainly enjoyed moderate to vigorous intensity sports-type exercise, while women mainly performed moderate-intensity aerobic exercise (data not shown). These patterns could be due to different motivations for exercise participation. For men, enjoyment and challenge are the main goals, while for women, appearance and weight loss are the major motivators [20,21]. For this reason, men tend to engage primarily in competitive sports, while women tend to engage in relatively safe and effective low-intensity to moderate-intensity aerobic exercise, such as walking, which does not require technical expertise [17,21].

The structural differences in CB networks according to gender could be explained by gender-based differences in substrate and metabolism during exercise. It has also been reported that men utilize more muscle glycogen, excrete more urea nitrogen, have lower lipid utilization, and have more carbohydrate and protein metabolism than women [22-25]. Several studies have suggested that these gender-based differences in substrate and metabolism may be mediated by estrogen [25-28]. Previous studies have reported that glucose levels changed less during exercise in women than in men and that women depend more on fat sources for fuel utilization during exercise than men, which our results support [29-32].

Information about regular exercise was obtained using a self-reported questionnaire. This may have caused recall bias, but since the yes/no questions were used to assess whether an individual exercised regularly enough to sweat rather than to assess the frequency or average time spent exercising, recall bias would have been minimal. The use of dichotomous variables for regular exercise participation may also be a limitation. Although the frequency and average time of regular exercise were also investigated, using more intuitive information (a binary variable) was possible to secure the ideal combination of exercise behavior changes and the ease of interpretation.

We used CBs related to CMDs to examine the effect of changes in exercise behavior, but the actual risk of the incidence of CMDs could not be confirmed. Moreover, the Ansan-Ansung cohort used here included 10 follow-up studies, but we could use only 3 time points (third to fifth phases) to prevent excessive loss of subjects due to missing values. Therefore, the incidence of CMDs, such as type 2 diabetes, hypertension, and dyslipidemia, was very low during the 4 years between the third and fifth phases, which limited our ability to examine associations with changes in exercise behavior.

Despite some limitations, we divided the behavioral changes of regular exercise into 4 categories to comprehensively examine their effects on CBs. In addition, we not only suggested simple associations, as in conventional epidemiological studies, but we also showed the relationships of CBs in an integrated way through networks. Through a topological comparison analysis according to exercise patterns, we confirmed different relationships among CBs according to changes in exercise behavior and suggested potential mechanisms that account for the differences between men and women.

The CBs showed different patterns of changes associated with changes in exercise behavior. Consistent exercise or exercise-initiating behavior had favorable effects on CBs related to CMDs. This study’s findings suggest that those who exercise regularly should avoid becoming inactive and that sedentary individuals should be encouraged to become physically active.

## Figures and Tables

**Figure f4-epih-45-e2023026:**
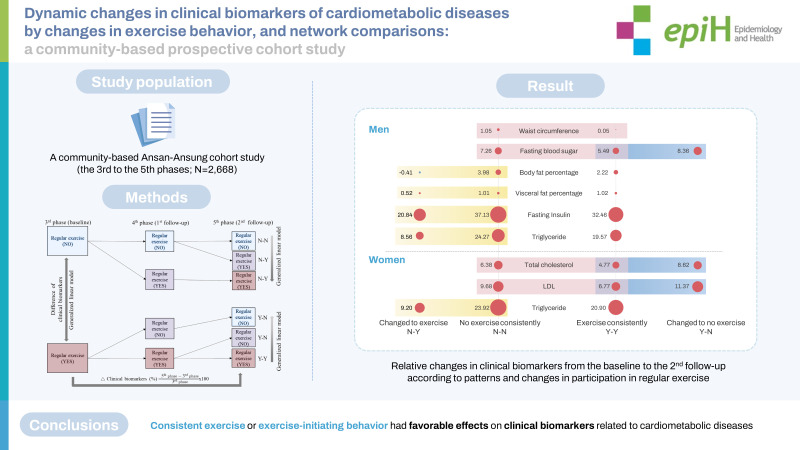


**Table 1.** Characteristics of the study population at baseline (third phase) by participation in regular exercise^1^

Characteristics		Participation in regular exercise					
		Men			Women		
		No (n=1,169) Reference	Yes (n=716)	OR (95% CI)	No (n=1,344) Reference	Yes (n=733)	OR (95% CI)
Age, mean±SD (yr)		55.3±8.61	52.4±7.55		54.9±8.67	52.0±7.34	
	40-49	33.6	47.4	1.00 (reference)	36.8	49.4	1.00 (reference)
	50-59	34.9	34.5	0.93 (0.74, 1.18)	31.6	33.6	0.86 (0.65, 1.13)
	≥60	31.5	15.2	0.79 (0.57, 1.08)	31.6	17.1	0.71 (0.49, 1.03)
Education							
	≤Middle school	46.7	25.4	1.00 (reference)	60.3	47.1	1.00 (reference)
	High school	36.4	44.4	1.65 (1.29, 2.11)	25.7	40.5	1.25 (0.98, 1.59)
	≥College	13.7	29.8	2.09 (1.51, 2.88)	5.5	9.3	1.29 (0.86, 1.94)
	Unknown	3.3	0.4		8.5	3.1	
Income (104 Korean won)							
	<200	53.8	29.8	1.00 (reference)	64.4	42.7	1.00 (reference)
	200-400	33.1	43.3	1.58 (1.22, 2.04)	25.5	37.7	1.50 (1.17, 1.91)
	≥400	12.4	26.7	2.02 (1.46, 2.79)	9.2	18.4	1.97 (1.42, 2.73)
	Unknown	0.7	0.3		1.0	1.2	
Marital status							
	Living with spouse	95.7	97.8	1.00 (reference)	83.7	88.7	1.00 (reference)
	Living alone	4.1	2.2	0.72 (0.39, 1.33)	16.2	11.1	0.98 (0.72, 1.34)
	Unknown	0.2	0.0		0.2	0.3	
Current occupation							
	Office	20.4	42.2	1.00 (reference)	7.2	9.0	1.00 (reference)
	Manual	72.0	48.5	0.64 (0.49, 0.83)	57.6	29.7	0.60 (0.41, 0.88)
	Unemployed/Homemaker	6.8	9.1	1.68 (1.08, 2.63)	32.1	59.6	1.85 (1.29, 2.66)
	Military/etc.	0.8	0.1	0.16 (0.02, 1.32)	3.1	1.6	0.54 (0.26, 1.13)
	Unknown	0.0	0.1				
Body mass index (kg/m^2^)							
	<18.5	3.6	1.1	0.49 (0.21, 1.11)	1.9	0.8	0.72 (0.28, 1.85)
	18.5-23.0	37.9	28.8	1.00 (reference)	35.6	32.1	1.00 (reference)
	23.0-25.0	29.2	32.0	1.20 (0.93, 1.54)	26.0	30.2	1.30 (1.02, 1.67)
	25.0-30.0	27.9	36.5	1.31 (1.02, 1.68)	32.1	33.7	1.27 (1.00, 1.62)
	≥30.0	1.5	1.7	1.18 (0.52, 2.67)	4.3	3.3	1.10 (0.65, 1.86)
Smoking							
	Never	24.6	29.3	1.00 (reference)	96.7	97.5	1.00 (reference)
	Former	30.4	40.9	1.02 (0.79, 1.32)	0.8	0.8	0.86 (0.30, 2.50)
	Current	44.9	29.8	0.57 (0.44, 0.74)	2.5	1.6	0.63 (0.31, 1.30)
	Unknown	0.1	0.0				
Drinking							
	Never	21.4	19.3	1.00 (reference)	72.7	63.3	1.00 (reference)
	Former	7.0	7.5	1.08 (0.70, 1.68)	1.6	1.5	0.75 (0.35, 1.64)
	Current	71.6	73.2	1.07 (0.83, 1.39)	25.7	35.2	1.56 (1.26, 1.93)
Menopause status							
	Pre-menopause	-	-	-	37.5	47.1	1.00 (reference)
	Post-menopause	-	-	-	62.1	52.5	1.19 (0.91, 1.57)
	Unknown	-	-	-	0.5	0.4	-

Values are presented as %.

1 Results from logistic regression model; regular exercise at the third phase (binary)=age (category)+education level (category)+income (category) +marital status (category)+current occupation (category)+body mass index (category)+smoking (category)+drinking habit (category), (+menopause status in women).

**Table 2.** Distribution of biomarkers at baseline (third phase) by participation in regular exercise^1^

Variables	Regular exercise at third phase			
	Men		Women	
	No (n=1,169)	Yes (n=716)	No (n=1,169)	Yes (n=716)
SBP (mmHg)	106.91 (98.56, 115.26)	105.92 (97.54, 114.30)	110.10 (104.81, 115.40)	110.11 (104.80, 115.42)
DBP (mmHg)	70.82 (65.07, 76.58)	70.16 (64.38, 75.94)	73.34 (69.78, 76.90)	73.09 (69.52, 76.66)
Waist circumference (cm)	82.89 (79.72, 86.05)^*^	82.02 (78.84, 85.19)^*^	78.53 (76.13, 80.92)^*^	77.34 (74.94, 79.75)^*^
Waist hip ratio	0.93 (0.89, 0.96)^*^	0.91 (0.88, 0.95)^*^	0.86 (0.83, 0.89)^*^	0.84 (0.81, 0.87)^*^
Body fat percentage (%)	20.54 (18.15, 22.93)	20.31 (17.91, 22.71)	29.90 (28.51, 31.28)	29.67 (28.28, 31.06)
Visceral fat percentage (%)	0.91 (0.89, 0.92)^*^	0.90 (0.89, 0.92)^*^	0.90 (0.89, 0.91)^*^	0.90 (0.88, 0.91)^*^
Obesity degree (%)	113.58 (111.55, 115.60)	113.46 (111.43, 115.49)	119.74 (118.37, 121.12)	119.47 (118.09, 120.85)
Fasting blood sugar (mg/dL)	94.01 (87.10, 100.91)	93.55 (86.62, 100.48)	87.06 (83.37, 90.75)	86.88 (83.18, 90.58)
HbA1c (%)	5.22 (4.93, 5.51)	5.23 (4.94, 5.53)	5.44 (5.28, 5.61)	5.42 (5.25, 5.59)
Fasting insulin (μIU/mL)	6.97 (4.36, 9.57)	6.68 (4.06, 9.30)	9.19 (7.67, 10.72)^*^	8.80 (7.28, 10.33)^*^
Total cholesterol (mg/dL)	180.72 (155.57, 205.86)	180.68 (155.44, 205.92)	201.63 (186.79, 216.46)^*^	205.86 (190.98, 220.73)^*^
HDL cholesterol (mg/dL)	34.73 (26.87, 42.59)^*^	36.15 (28.26, 44.05)^*^	47.35 (42.76, 51.93)^*^	48.70 (44.10, 53.29)^*^
LDL cholesterol (mg/dL)	120.77 (96.78, 144.76)	121.37 (97.28, 145.45)	134.99 (121.71, 148.28)^*^	138.38 (125.06, 151.70)^*^
Triglyceride (mg/dL)	126.12 (50.76, 201.49)	115.80 (40.15, 191.46)	96.45 (65.61, 127.28)	93.88 (62.96, 124.80)

Values are presented as LSMean (95% confidence interval). LSMean, least-squares mean; SBP, systolic blood pressure; DBP, diastolic blood pressure; HbA1c, hemoglobin A1c; HDL, high-density lipoprotein; LDL, low-density lipoprotein.

1 LSMeans were estimated by generalized linear model in men and women, respectively; dependent variable: each clinical biomarker; independent variable=regular exercise; covariates: age, education, income, marital status, job, body mass index, smoking, and drinking.

* p<0.05 results from the generalized linear model.

**Table 3.** Relative changes^1^ in biomarkers between baseline (third phase) and at the second follow-up (fifth phase) in men and women, according to patterns of participation in regular exercise

Variables		Patterns of participation in regular exercise			
		Change to exercise behavior	No exercise consistently	Regular exercise consistently	Change to no-exercise behavior
Men		N-Y (n=181)	N-N (n=576)	Y-Y (n=332)	Y-N (n=183)
	SBP (mmHg)	5.32 (3.74, 6.90)	4.99 (4.09, 5.89)	5.67 (4.47, 6.86)	4.86 (3.36, 6.35)
	DBP (mmHg)	3.00 (1.49, 4.52)	2.85 (1.97, 3.72)	3.65 (2.49, 4.81)	4.39 (2.84, 5.95)
	Waist circumference (cm)	0.38 (-0.41, 1.16)	1.05 (0.63, 1.47)	0.05 (-0.51, 0.62)^3^	0.66 (-0.06, 1.39)
	Waist hip ratio	0.85 (0.15, 1.55)	1.06 (0.69, 1.44)	0.57 (0.08, 1.07)	1.20 (0.62, 1.78)
	Body fat percentage (%)	-0.41 (-2.72, 1.89)^2^	4.03 (2.77, 5.28)	2.22 (0.55, 3.88)	3.47 (1.45, 5.49)
	Visceral fat percentage (%)	0.52 (0.20, 0.83)^2^	1.03 (0.85, 1.21)	1.02 (0.79, 1.26)	1.20 (0.88, 1.52)
	Obesity degree (%)	-0.27 (-0.94, 0.40)	0.40 (0.02, 0.77)	0.39 (-0.11, 0.88)	0.44 (-0.20, 1.07)
	Fasting blood sugar (mg/dL)	7.03 (5.27, 8.79)	7.26 (6.37, 8.14)	5.53 (4.36, 6.70)^3^	8.36 (6.82, 9.91)^4^
	HbA1c (%)	4.31 (3.27, 5.35)	3.42 (2.86, 3.99)	2.88 (2.14, 3.63)	4.15 (3.10, 5.20)
	Fasting insulin (μIU/mL)	20.84 (9.13, 32.56)^2^	36.90 (30.51, 43.30)	32.46 (23.99, 40.93)	45.29 (34.80, 55.79)
	Total cholesterol (mg/dL)	3.67 (1.33, 6.02)	4.69 (3.43, 5.96)	4.28 (2.60, 5.95)	4.28 (2.17, 6.40)
	HDL cholesterol (mg/dL)	0.70 (-2.33, 3.73)	0.14 (-1.53, 1.81)	1.34 (-0.87, 3.55)	0.05 (-2.72, 2.82)
	LDL cholesterol (mg/dL)	-9.30 (-29.27, 10.66)	6.68 (3.15, 10.21)	6.87 (2.19, 11.55)	11.36 (6.33, 16.38)
	Triglyceride (mg/dL)	8.56 (-0.83, 17.95)^2^	24.53 (19.31, 29.75)	19.57 (12.65, 26.49)	15.95 (7.92, 23.97)
Women		N-Y (n=241)	N-N (n=645)	Y-Y (n=303)	Y-N (n=207)
	SBP (mmHg)	5.12 (3.57, 6.67)	6.68 (5.73, 7.62)	5.66 (4.27, 7.06)	4.33 (2.82, 5.85)
	DBP (mmHg)	2.84 (1.36, 4.32)	4.57 (3.67, 5.47)	3.29 (1.97, 4.61)	3.56 (2.10, 5.01)
	Waist circumference (cm)	0.04 (-0.71, 0.78)	0.15 (-0.29, 0.59)	0.24 (-0.41, 0.88)	0.60 (-0.16, 1.36)
	Waist hip ratio	-0.46 (-1.19, 0.28)	-0.13 (-0.54, 0.28)	-0.11 (-0.72, 0.50)	0.62 (-0.04, 1.28)
	Body fat percentage (%)	1.32 (-0.07, 2.71)	2.03 (1.15, 2.92)	0.68 (-0.62, 1.98)	2.27 (0.79, 3.75)
	Visceral fat percentage (%)	0.87 (0.55, 1.18)	0.87 (0.66, 1.07)	0.86 (0.56, 1.16)	1.24 (0.86, 1.61)
	Obesity degree (%)	0.01 (-0.63, 0.65)	-0.13 (-0.54, 0.28)	0.11 (-0.49, 0.71)	0.78 (0.02, 1.54)
	Fasting blood sugar (mg/dL)	5.71 (4.51, 6.92)	6.43 (5.68, 7.19)	6.08 (4.96, 7.19)	6.40 (5.16, 7.64)
	HbA1c (%)	5.17 (4.09, 6.25)	4.01 (3.47, 4.56)	4.34 (3.54, 5.14)	4.71 (3.82, 5.60)
	Fasting insulin (μIU/mL)	24.94 (15.90, 33.98)	27.76 (21.87, 33.65)	32.48 (23.80, 41.16)	24.81 (15.42, 34.21)
	Total cholesterol (mg/dL)	4.12 (2.03, 6.21)	6.38 (5.10, 7.66)	3.94 (2.06, 5.83)^3^	8.62 (6.51, 10.72)^4^
	HDL cholesterol (mg/dL)	0.22 (-2.22, 2.67)	-0.25 (-1.73, 1.24)	-0.47 (-2.65, 1.72)	-0.30 (-2.82, 2.21)
	LDL cholesterol (mg/dL)	7.93 (4.67, 11.18)	9.68 (7.69, 11.67)	5.81 (2.88, 8.74)^3^	11.37 (8.04, 14.70)^4^
	Triglyceride (mg/dL)	9.20 (1.94, 16.46)^2^	24.17 (19.62, 28.73)	20.90 (14.19, 27.60)	28.98 (21.04, 36.92)

Values are presented as LSMean (95% confidence interval). LSMean, least-squares mean; SBP, systolic blood pressure; DBP, diastolic blood pressure; HbA1c, hemoglobin A1c; HDL, high-density lipoprotein; LDL, low-density lipoprotein.

1 LSMeans of relative changes (%) were estimated by generalized linear model; dependent variable: each clinical biomarker; independent variable=patterns of regular exercise; covariate: age.

2 Significant decrease or a smaller increase in N-Y than in N-N (p<0.05 from generalized linear model).

3 Significantly lower increase in Y-Y than in N-N (p<0.05 from generalized linear model).

4 Significantly greater increase in Y-N than in Y-Y (p<0.05 from generalized linear model).

## SUPPLEMENTARY MATERIALS

Supplementary Material 1. Study scheme, and categorization of change patterns in regular exercise behavior (Ansan-Ansung cohort study)

Supplementary Material 2. Characteristics of the study population at baseline (3rd phase) by sex

Supplementary Material 3. Distribution of- biomarkers by sex at baseline (3rd phase)

Supplementary Material 4. Characteristics of study population at baseline (3rd phase) by patterns of participation in regular exercise
