# Supplementary material for: Dynamic changes in clinical biomarkers of cardiometabolic diseases by changes in exercise behavior, and network comparisons: a community-based prospective cohort study in Korea
Source: Epidemiol Health. 2023 Feb 16;45:e2023026. doi: 10.4178/epih.e2023026 (PMC10396801; doi:10.4178/epih.e2023026)
Supplement: Supplementary Material 1. — Study scheme, and categorization of change patterns in regular exercise behavior (Ansan-Ansung cohort study) [file epih-45-e2023026-Supplementary-1.docx]

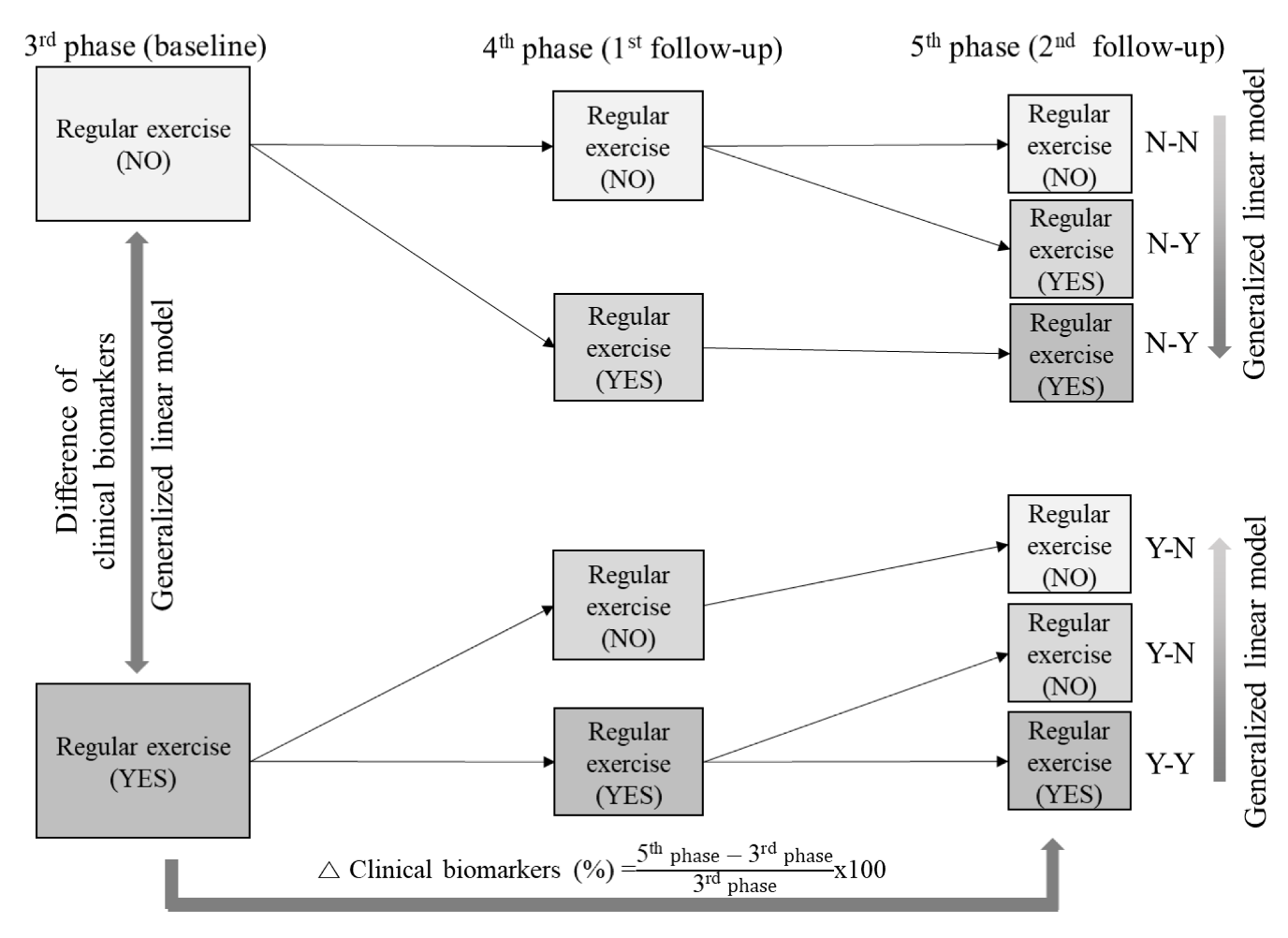


Supplementary Material 1. Study scheme, and categorization of change patterns in regular exercise behavior (Ansan-Ansung cohort study
