# Supplementary material for: Dynamic changes in clinical biomarkers of cardiometabolic diseases by changes in exercise behavior, and network comparisons: a community-based prospective cohort study in Korea
Source: Epidemiol Health. 2023 Feb 16;45:e2023026. doi: 10.4178/epih.e2023026 (PMC10396801; doi:10.4178/epih.e2023026)
Supplement: Supplementary Material 2. — Characteristics of the study population at baseline (3rd phase) by sex [file epih-45-e2023026-Supplementary-2.docx]

Supplementary Material 2. Characteristics of the study population at baseline (3^rd^ phase) by sex

|  | Total | | Men | | Women | |  |
| --- | --- | --- | --- | --- | --- | --- | --- |
|  | N=3,962 | | N=1,885 | | N=2,077 | |  |
|  | N | (%) | N | (%) | N | (%) | p-value |
| Age, Mean ± SD | 54.1 ± 8.34 | | 54.2 ± 8.34 | | 53.9 ± 8.34 | | 0.1693 ^a^ |
| 40-49 | 1589 | (40.1) | 732 | (38.8) | 857 | (41.3) | 0.1934 ^b^ |
| 50-59 | 1,325 | (33.4) | 655 | (34.8) | 670 | (32.3) |  |
| 60 + | 1,048 | (26.5) | 498 | (26.4) | 550 | (26.5) |  |
| Education |  |  |  |  |  |  |  |
| ≤Middle school | 1,884 | (47.6) | 728 | (38.6) | 1,156 | (55.7) | <0.0001 ^b^ |
| High school | 1,385 | (35.0) | 743 | (39.4) | 642 | (30.9) |  |
| ≥College | 515 | (13.0) | 373 | (19.8) | 142 | (6.8) |  |
| Unknown | 178 | (4.5) | 41 | (2.2) | 137 | (6.6) |  |
| Income (₩10,000) |  |  |  |  |  |  |  |
| <200 | 2,020 | (51.0) | 842 | (44.7) | 1,178 | (56.7) | <0.0001 ^b^ |
| 200-400 | 1,315 | (33.2) | 697 | (37.0) | 618 | (29.8) |  |
| ≥400 | 594 | (15.0) | 336 | (17.8) | 258 | (12.4) |  |
| Unknown | 33 | (0.8) | 10 | (0.5) | 23 | (1.1) |  |
| Marital status |  |  |  |  |  |  |  |
| Living with spouse | 3,594 | (90.7) | 1,819 | (96.5) | 1,775 | (85.5) | <0.0001 ^b^ |
| Living alone | 362 | (9.1) | 64 | (3.4) | 298 | (14.4) |  |
| Unknown | 6 | (0.2) | 2 | (0.1) | 4 | (0.2) |  |
| Current occupation |  |  |  |  |  |  |  |
| Office | 703 | (17.7) | 540 | (28.7) | 163 | (7.9) | <0.0001 ^b^ |
| Manual | 2,181 | (55.1) | 1,189 | (63.1) | 992 | (47.8) |  |
| Unemployed/House wives | 1,014 | (25.6) | 145 | (7.7) | 869 | (41.8) |  |
| Soldier/etc. | 63 | (1.6) | 10 | (0.5) | 53 | (2.6) |  |
| Unknown | 1 | (0.0) | 1 | (0.1) | 0 | (0.0) |  |
| BMI |  |  |  |  |  |  |  |
| <18.5 | 82 | (2.1) | 50 | (2.7) | 32 | (1.5) | <0.0001 ^b^ |
| 18.5-23 | 1,363 | (34.4) | 649 | (34.4) | 714 | (34.4) |  |
| 23-25 | 1,140 | (28.8) | 570 | (30.2) | 870 | (27.4) |  |
| 25-30 | 1,266 | (32.0) | 587 | (31.1) | 679 | (32.7) |  |
| ≥30 | 111 | (2.8) | 29 | (1.5) | 82 | (4.0) |  |
|  |  |  |  |  |  |  |  |
| Smoking |  |  |  |  |  |  |  |
| Never | 2,513 | (63.4) | 498 | (26.4) | 2,015 | (97.0) | <0.0001 ^b^ |
| Former | 665 | (16.8) | 648 | (34.4) | 17 | (0.8) |  |
| Current | 783 | (19.8) | 738 | (39.2) | 45 | (2.2) |  |
| Unknown | 1 | (0.0) | 1 | (0.1) | 0 | (0.0) |  |
| Drinking |  |  |  |  |  |  |  |
| Never | 1,829 | (46.2) | 388 | (20.6) | 1,441 | (69.4) | <0.0001 ^b^ |
| Former | 168 | (4.2) | 136 | (7.2) | 32 | (1.5) |  |
| Current | 1,965 | (49.6) | 1,361 | (72.2) | 604 | (29.1) |  |
| Regular exercise |  |  |  | |  | |  |
| No | 2,513 | (63.4) | 1,169 | (62.0) | 1,344 | (64.7) | 0.0788 ^b^ |
| Yes | 1,449 | (36.6) | 716 | (38.0) | 733 | (35.3) |  |

^a^ Wilcoxon rank-sum test

^b^ Chi-square test
