# Supplementary material for: Dynamic changes in clinical biomarkers of cardiometabolic diseases by changes in exercise behavior, and network comparisons: a community-based prospective cohort study in Korea
Source: Epidemiol Health. 2023 Feb 16;45:e2023026. doi: 10.4178/epih.e2023026 (PMC10396801; doi:10.4178/epih.e2023026)
Supplement: Supplementary Material 3. — Distribution of- biomarkers by sex at baseline (3rd phase) [file epih-45-e2023026-Supplementary-3.docx]

Supplementary Material 3. Distribution of- biomarkers by sex at baseline (3^rd^ phase)

|  | Men | | | |  | Women | | | |  |
| --- | --- | --- | --- | --- | --- | --- | --- | --- | --- | --- |
|  | N=1,885 | | | |  | N=2,077 | | | |  |
|  | Mean | (SD) | Median | (Q1 – Q3) |  | Mean | (SD) | Median | (Q1 – Q3) | p-value ^a^ |
| SBP | 111.04 | (11.00) | 110 | (103-119) |  | 108.58 | (12.28) | 108 | (99-117) | <0.0001 |
| DBP | 75.70 | (7.44) | 76 | (70-81) |  | 72.80 | (8.15) | 73 | (68-79) | <0.0001 |
| Waist circumference | 83.50 | (7.40) | 83.5 | (78.8-88.3) |  | 81.52 | (9.05) | 81 | (75-87.2) | <0.0001 |
| Waist hip ratio | 0.91 | (0.06) | 0.91 | (0.88-0.95) |  | 0.89 | (0.09) | 0.89 | (0.83-0.95) | <0.0001 |
| Body fat percentage | 20.02 | (4.75) | 19.9 | (16.7-23.3) |  | 30.02 | (5.19) | 30.4 | (26.5-33.5) | <0.0001 |
| Visceral fat percentage | 0.90 | (0.04) | 0.9 | (0.87-0.92) |  | 0.90 | (0.05) | 0.9 | (0.87-0.93) | 0.0500 |
| Obesity degree | 111.47 | (13.04) | 112 | (103-120) |  | 119.85 | (15.23) | 118 | (109-129) | <0.0001 |
| Fasting blood sugar | 91.25 | (8.95) | 90 | (85-96) |  | 87.70 | (8.19) | 87 | (82-92) | <0.0001 |
| HbA1c | 5.38 | (0.38) | 5.4 | (5.1-5.6) |  | 5.41 | (0.37) | 5.4 | (5.2-5.6) | 0.0101 |
| Fasting insulin | 6.77 | (3.43) | 6.1 | (4.9-7.9) |  | 7.51 | (3.39) | 6.9 | (5.6-8.6) | <0.0001 |
| Total cholesterol | 186.77 | (32.35) | 185 | (164-207) |  | 192.83 | (32.92) | 190 | (170-213) | <0.0001 |
| HDL | 43.56 | (10.55) | 42 | (36-49) |  | 46.30 | (10.07) | 45 | (39-52) | <0.0001 |
| LDL | 115.63 | (30.80) | 115 | (97.2-135.6) |  | 123.67 | (29.22) | 121.8 | (103.8-142.2) | <0.0001 |
| Triglyceride | 137.85 | (97.41) | 114 | (81-164) |  | 114.32 | (67.89) | 98 | (72-137) | <0.0001 |

^a^ Wilcoxon rank-sum test
