# Supplementary material for: Dynamic changes in clinical biomarkers of cardiometabolic diseases by changes in exercise behavior, and network comparisons: a community-based prospective cohort study in Korea
Source: Epidemiol Health. 2023 Feb 16;45:e2023026. doi: 10.4178/epih.e2023026 (PMC10396801; doi:10.4178/epih.e2023026)
Supplement: Supplementary Material 4. — Characteristics of study population at baseline (3rd phase) by patterns of participation in regular exercise [file epih-45-e2023026-Supplementary-4.docx]

Supplementary Material 4. Characteristics of study population at baseline (3^rd^ phase) by patterns of participation in regular exercise

|  | Men | | | |  | Women | | | |  |
| --- | --- | --- | --- | --- | --- | --- | --- | --- | --- | --- |
|  | No exercise consistently | | Regular exercise consistently | |  | No exercise consistently | | Regular exercise consistently | |  |
|  | N=576 | | N=332 | |  | N=645 | | N=303 | |  |
|  | N | (%) | N | (%) | p-value | N | (%) | N | (%) | p-value |
| Age, Mean ± SD | 55.7 ± 8.60 | | 52.2 ± 7.16 | | <0.0001 ^a^ | 55.7 ± 8.74 | | 51.6 ± 6.81 | | <0.0001 ^a^ |
| 40-49 | 180 | (31.3) | 158 | (47.6) | <0.0001 ^b^ | 211 | (32.7) | 149 | (49.2) | <0.0001 ^b^ |
| 50-59 | 199 | (34.6) | 121 | (36.5) |  | 203 | (31.5) | 108 | (35.6) |  |
| 60 + | 197 | (34.2) | 53 | (16.0) |  | 231 | (35.8) | 46 | (15.2) |  |
| Education |  |  |  |  |  |  |  |  |  |  |
| ≤Middle school | 285 | (49.5) | 64 | (19.3) | <0.0001 ^b^ | 402 | (62.3) | 136 | (44.9) | <0.0001 ^b^ |
| High school | 207 | (35.9) | 144 | (43.4) |  | 155 | (24.0) | 130 | (42.9) |  |
| ≥College | 69 | (12.0) | 124 | (37.4) |  | 30 | (4.7) | 35 | (11.6) |  |
| Unknown | 15 | (2.6) | 0 | (0.0) |  | 58 | (9.0) | 2 | (0.7) |  |
| Income(₩10,000) |  |  |  |  |  |  |  |  |  |  |
| <200 | 335 | (58.2) | 82 | (24.7) | <0.0001 ^b^ | 430 | (66.7) | 124 | (40.9) | <0.0001 ^b^ |
| 200-400 | 177 | (30.7) | 146 | (44.0) |  | 157 | (24.3) | 116 | (38.3) |  |
| ≥400 | 60 | (10.4) | 103 | (31.0) |  | 49 | (7.6) | 60 | (19.8) |  |
| Unknown | 4 | (0.7) | 1 | (0.3) |  | 9 | (1.4) | 3 | (1.0) |  |
| Marital status |  |  |  |  |  |  |  |  |  |  |
| Living with spouse | 549 | (95.3) | 325 | (97.9) | 0.0487 ^b^ | 537 | (83.3) | 276 | (91.1) | 0.0015 ^b^ |
| Living alone | 27 | (4.7) | 7 | (2.1) |  | 107 | (16.6) | 27 | (8.9) |  |
| Unknown |  |  |  |  |  | 1 | (0.2) | 0 | (0.0) |  |
|  |  |  |  |  |  |  |  |  |  |  |
|  |  |  |  |  |  |  |  |  |  |  |
| Current occupation |  |  |  |  |  |  |  |  |  |  |
| Office | 91 | (15.8) | 167 | (50.3) | <0.0001 ^b^ | 42 | (6.5) | 29 | (9.6) | <0.0001 ^b^ |
| Manual | 448 | (77.8) | 137 | (41.3) |  | 396 | (61.4) | 82 | (27.1) |  |
| Unemployed/House wives | 32 | (5.6) | 27 | (8.1) |  | 192 | (29.8) | 187 | (61.7) |  |
| Soldier/etc. | 5 | (0.9) | 0 | (0.0) |  | 15 | (2.3) | 5 | (1.7) |  |
| Unknown | 0 | (0.0) | 1 | (0.3) |  |  |  |  |  |  |
| BMI |  |  |  |  |  |  |  |  |  |  |
| <18.5 | 24 | (4.2) | 4 | (1.2) | <0.0001 ^b^ | 12 | (1.9) | 3 | (1.0) | 0.2362 ^b^ |
| 18.5-23 | 235 | (40.8) | 83 | (25.0) |  | 232 | (36.0) | 95 | (31.4) |  |
| 23-25 | 149 | (25.9) | 114 | (34.3) |  | 158 | (24.5) | 93 | (30.7) |  |
| 25-30 | 160 | (27.8) | 127 | (38.3) |  | 217 | (33.6) | 102 | (33.7) |  |
| ≥30 | 8 | (1.4) | 4 | (1.2) |  | 26 | (4.0) | 10 | (3.3) |  |
| Smoking |  |  |  |  |  |  |  |  |  |  |
| Never | 139 | (24.1) | 107 | (32.2) | <0.0001 ^b^ | 633 | (98.1) | 296 | (97.7) | 0.5777 ^b^ |
| Former | 167 | (29.0) | 151 | (45.5) |  | 4 | (0.6) | 1 | (0.3) |  |
| Current | 269 | (46.7) | 74 | (22.3) |  | 8 | (1.2) | 6 | (2.0) |  |
| Unknown | 1 | (0.2) | 0 | (0.0) |  |  |  |  |  |  |
| Drinking |  |  |  |  |  |  |  |  |  |  |
| Never | 123 | (21.4) | 57 | (17.2) | 0.1509 ^b^ | 485 | (75.2) | 190 | (62.7) | 0.0004 ^b^ |
| Former | 41 | (7.1) | 18 | (5.4) |  | 8 | (1.2) | 5 | (1.7) |  |
| Current | 412 | (71.5) | 257 | (77.4) |  | 152 | (23.6) | 108 | (35.6) |  |
| Menopause status |  |  |  |  |  |  |  |  |  |  |
| Pre-menopause |  |  |  |  |  | 47 | (7.3) | 29 | (9.6) | 0.0794 ^b^ |
| Changed pre to post |  |  |  |  |  | 26 | (4.0) | 22 | (7.3) |  |
| Post menopause |  |  |  |  |  | 231 | (35.8) | 103 | (34.0) |  |
| Unknown |  |  |  |  |  | 341 | (52.9) | 149 | (49.2) |  |

^a^ Wilcoxon rank-sum test

^b^ Chi-square test
